# Predictors of Futile Inter-Hospital Transfer for Endovascular Thrombectomy in Anterior Circulation Acute Ischemic Stroke Due to Large Vessel Occlusion

**DOI:** 10.3390/brainsci15121320

**Published:** 2025-12-11

**Authors:** Tushar B. Patil, Aviraj Satish Deshmukh, Zacharie Gagné, Christine Hawkes, Aris H. Katsanos, Naif Faisal Alharbi, Mohammed Mesfer Alwadai, Rhonda McNicolle-White, Mukul Sharma, Brian van Adel

**Affiliations:** 1 Department of Neurology, Jawaharlal Nehru Medical College, Sawangi, Wardha 442005, India; tushar.neuro@gmail.com; 2 Division of Clinical Sciences, Health Sciences North, Northern Ontario School of Medicine, Sudbury, ON P3E 2C6, Canada; zgagne@nosm.ca; 3 Division of Neurology, Department of Medicine, Sunnybrook Health Sciences Centre, Temerty School of Medicine, University of Toronto, Toronto, ON M5S 3H2, Canada; christine.hawkes@sunnybrook.ca; 4 Division of Neurology, Hamilton General Hospital, McMaster University, Hamilton, ON L8S 4L8, Canada; 5 Department of Neurology, King Fahd General Hospital, Ministry of Health, Jeddah 23325, Saudi Arabiamohammed.alwadai@medportal.ca (M.M.A.); 6 Department of Neurointervention, Hamilton General Hospital, McMaster University, Hamilton, ON L8S 4L8, Canada; mcnicolr@hhsc.ca (R.M.-W.);

**Keywords:** EVT, ischemic stroke, LVO, inter-hospital transfer, futile transfer

## Abstract

**Background:** Endovascular therapy (EVT) is a standard treatment for acute ischemic stroke (AIS) with large vessel occlusion (LVO), but inter-hospital transfers from primary stroke centers (PSCs) to comprehensive stroke centers (CSCs) can result in delayed treatment and worse outcomes. Up to 30–40% of patients transferred may not receive EVT. This study investigates the causes of futile transfers to a CSC in Canada, aiming to identify its predictors. **Methods:** We conducted a retrospective analysis of consecutive patients transferred for EVT between 1 April 2017 and 31 December 2020, from PSCs and community hospitals (CH) to a CSC in an urban area of Canada. Data on demographics, clinical characteristics, and treatment outcomes were collected. Descriptive and comparative analyses were performed to identify factors contributing to non-receipt of EVT. **Results:** Of the transferred 326 patients, 241 (73.9%) underwent EVT, and 85 (26%) did not. The main reasons for not performing EVT were recanalization of the target vessel (44.7%), infarct growth (29.4%), clinical improvement or low NIHSS (17.6%), and hemorrhagic transformation (8.2%). Predictors of futility were lower NIHSS at presentation, intravenous thrombolysis (IVT) at the PSC, and greater ASPECTS decay during transport. **Conclusions:** Our study concluded that 26% of inter-hospital transfers for EVT were futile, primarily due to infarct growth, recanalization of the target vessel, and low NIHSS. These findings suggest that closer monitoring of clinical status, consideration of direct transfers to CSCs, and enhanced triage strategies may help reduce futile transfers and improve patient outcomes.

## 1. Introduction

Endovascular therapy (EVT) has rapidly evolved and been adopted as the standard of care for the management of acute ischemic stroke (AIS). The time window for EVT has been extended up to 24 h from the last known normal (LKN) for patients meeting specific imaging criteria (LVO) [1]. Delivery of EVT services is hampered by its availability. Hospitals without the capability to perform EVT, such as most Primary Stroke Centers (PSCs) and community hospitals (CH), must, therefore, transfer patients to CSCs for EVT. While these inter-hospital transfers are feasible and safe, they are associated with treatment delay and hence worse clinical outcomes, as they allow for greater infarct growth while patients await treatment [2,3,4,5]. Despite best efforts, inter-hospital transfers for LVO are futile in up to 30–40% patients due to various patient-related and local elements [4]. Understanding these factors could help to mitigate unnecessary transfers. Our aim is to determine the major causes for not performing EVT in patients with LVO following inter-hospital land transfer in a Canadian CSC situated in an urban area.

## 2. Materials and Methods

Institutional research ethics board (REB) approval was obtained for a retrospective analysis of a prospectively collected quality assurance stroke database. Consent was waived for the study due to its retrospective nature. Our CSC is the only center performing EVT in a Local Health Integration Network (LHIN) 3 and 4 region with a nearly 2.1 million people residing in it (Hamilton, Niagara, Haldimand, Brant & Waterloo Wellington region) [5]. Patients are transferred by a ground ambulance with a drive time of less than 60 min. We interface with 5 PSCs and 10 community hospitals for successful triage and transfer of patients for EVT. All acute ischemic stroke patients transferred to our hospital are entered into the quality assurance database.

Once the patient is deemed suitable for EVT at the peripheral hospital, the stroke neurologist and neurointerventionist at the comprehensive stroke center (CSC) are contacted using the CritiCall Ontario network. A conference call is initiated among the referring site team, the stroke neurologist, and the neurointerventionist for discussion and real-time image review. The Emergency Neuro Image Transfer System (ENITS) is used for viewing CT images by both specialists without waiting for a formal radiology report, thereby avoiding delays. The patients who were eligible for intravenous thrombolysis (IVT) were treated with an alteplase (tPA) dose of 0.9 mg/kg at the referring hospital. If the stroke neurologist and neurointerventionist deem the patient a candidate for thrombectomy, the stroke code group page is activated, and relevant team members aim to meet at the emergency department bay prior to the patient’s arrival. At our institution, the decision to transfer patients for EVT is generally based on a patient’s pre-stroke Modified Rankin Scale score (mRS), National Institutes of Health Stroke Scale (NIHSS) at the time of presentation, and imaging findings on computerized tomography (CT) scans confirming LVO, status of collateral circulation, and salvageable tissue. The usual time window for EVT was 6 h from LKN. Patients beyond 6 h from LKN were considered for EVT based on neuroimaging, after discussion between the stroke neurologist and neurointerventionist. CT angiography scans were obtained at all PSCs and some of the community hospitals when possible, without delaying inter-hospital transfer. The CT perfusion (CTP) facility was initiated at our center and PSCs after July 2019.

Data was collected for all the anterior circulation patients who were transferred for EVT between 1 April 2017 to 31 December 2020, with occlusions of the internal carotid artery or M1 and proximal M2 segments of the middle cerebral artery. Patient demographics, including age, sex, admission NIHSS, premorbid mRS score, site of intracranial occlusion, and intravenous thrombolysis (IVT), were collected. Exclusion criteria included patients who presented directly to a CSC, inpatients with stroke, and patients with posterior circulation stroke.

### 2.1. Analytical Framework

This is a descriptive study analyzing a retrospective cohort of patients who were transferred to a CSC for EVT. Baseline characters, IVT, various time intervals, NIHSS, and ASPECTS will be presented and compared between the patients who undergo EVT and those who do not undergo EVT. Multivariate logistic regression was performed to identify independent predictors. Most common causes of not performing EVT were identified, and characteristics of these subgroups were described.

### 2.2. Statistical Analysis

Continuous variables were presented as mean, standard deviation, median, and range. Categorical variables were presented as numbers and percentages. Normal distribution of data was determined by the Shapiro–Wilk test. The Chi-square test was used to test the difference between two groups for categorical data. Student’s t test for independent samples was used for analysis of means of two groups, for normally distributed data. The Mann–Whitney U test was used when the data was not normally distributed. Multivariate logistic regression was performed to assess factors independently associated with futile transfer. Statistical analysis was performed using Statistical Package for Social Sciences, version 16 for Windows (SPSS, Chicago, IL, USA).

## 3. Results

We studied patients who were transferred to our comprehensive stroke center between 2017 and 2020. In this time interval, 326 patients with anterior circulation acute ischemic stroke with LVO were received at the CSC. Out of these, 305 (93.5%) patients were transferred from PSCs, and 21 (6.4%) were transferred from community hospitals. After clinical and imaging evaluation, 241 (73.9%) patients underwent EVT, and 85 (26%) did not undergo EVT. Thus, the prevalence of futile inter-hospital transfers was 26% in our study. The comparison of characteristics of the study population in the two groups is presented in Table 1.

Average age (71 vs. 69.6), sex distribution (45.9 vs. 43.6 female %), and NIHSS at the presenting hospital (16 vs. 17) were similar in both groups. However, after transfer to CSC, the group that did not undergo EVT had significantly lower NIHSS (9 vs. 17). A significantly higher proportion of patients in the futile transfer group received IVT at the PSC (75.3 vs. 54.8%). Although patients in the futile transfer group presented significantly later at the PSC (231.9 vs. 166.2 min), they had shorter Door-In-Door-Out time compared to the EVT group (91.7 vs. 105 min). There was no significant difference in the transport time (49.8 vs. 47.2 min) and the time since stroke at CSC presentation between the two groups. Patients in the futile transfer group had higher ASPECTS values at PSC, but lower ASPECTS values by the time they reached the CSC. There was no significant difference in the ASCETS decay between the two groups during interfacility transfer. Table 2 shows the results of multivariate logistic regression analysis for independent predictors of futile inter-hospital transfer. It was observed that no factor was significantly associated with the futility of transfer.

**Figure 1:**
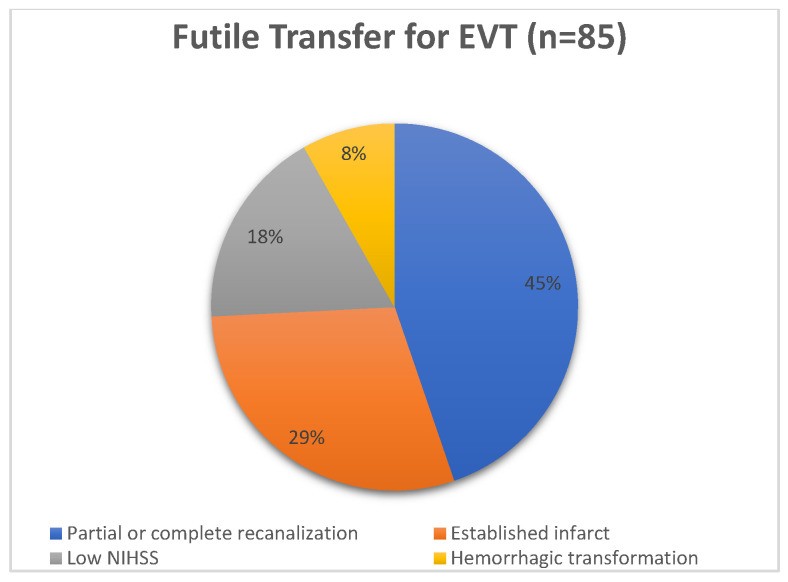
Percentage distribution of futile transfer.

Out of the 85 patients in the futile transfer group, the reasons for not performing EVT were the absence of LVO due to partial or complete recanalization or distal embolization in 38 (44.7%) patients, the presence of established infarct in 25 (29.4%) patients, clinical improvement (low NIHSS) in 15 (17.6%) patients, and hemorrhagic transformation of infarct post-IVT in 7 (8.2%) patients. The percentage distribution is shown in Figure 1.

The characteristics of the three main groups in the futile transfer cohort are provided in Table 3. As expected, patients in the established infarct category had higher NIHSS scores at the time of presentation to PSC, and deficits persisted by the time they reached CSC. The vast majority of patients in the recanalization category (89.4%) received thrombolysis. The Door-In-Door-Out (DIDO) time (time from admission to discharge at PSC) was higher in the recanalization group, likely due to the administration of thrombolysis. Transport times were similar in all three groups.

## 4. Discussion

In our study, patients were selected for transfer after discussion between the stroke neurologist, neurointerventionist, and referring site physician based on clinical and imaging criteria. EVT indication was reassessed on arrival at the CSC based on follow-up neuroimaging and clinical reassessment. Based on prevailing practice at the time this data was collected, all patients underwent repeat neuroimaging at the CSC. However, with the event of trials showing benefits of EVT even in patients with large infarct cores, this practice is undergoing change, and patients are considered for EVT without repeat neuroimaging. Patients were exclusively transferred by land. Between 1 April 2017 to 31 December 2020, 326 patients were transferred to our center for EVT. Out of these, 305 (93.5%) patients were transferred from PSCs, and 21 (6.4%) were transferred from community hospitals. Out of 326 patients, 85 patients (26%) did not undergo EVT after transfer. The most common reason for not performing the EVT was complete or partial recanalization of the target vessel (44.7%) noted on a follow-up scan at CSC. This was followed by established infarct in 29.4% and low NIHSS in 17.6% and a minority of the patients had hemorrhagic transformation. There were no demographic differences in patients who underwent EVT and who had a futile transfer. NIHSS, transfer time, and last known well to CSC arrival were similar in both groups. Administration of intravenous thrombolysis, ASPECT decay, and delayed presentation to PSC were the three main factors associated with futile transfer.

The neuronal injury following a large cerebral vessel occlusion is a dynamic process that depends on multiple factors. The decision to transfer a patient for EVT is taken at the PSC, depending upon the time window and tissue window at that time. The time window for EVT was 6 h from LKN. The tissue window was determined by a stroke neurologist and neurointerventionist after analysis of neuroimaging. However, by the time the patient reaches the CSC, the dynamics of the stroke process evolve, and some patients are not suitable candidates for EVT at the CSC. Based on the rate of progression of the infarct core, patients can be slow or fast progressors. There may be multiple factors which may be responsible for a rapid versus slow infarct progression. The status of collateral circulation is one important factor. In patients with better collateral circulation, the ischemic penumbra survives longer with a better opportunity for a good outcome following EVT. Similarly, evidence suggests that patients who have received intravenous thrombolysis (IVT) at PSC do better as compared to those who directly undergo EVT. This could be due to partial recanalization achieved by IVT, which may help slow down conversion of ischemic penumbra to the infarct core. The clinical outcome can also be altered due to medical complications during transfer to a CSC. Pallesen et al. studied 615 patients who were transferred for EVT at a CSC. There were no significant cardiopulmonary or medical complications during the transfer, thus establishing the safety of inter-hospital transfer for EVT [6]. However, the clinical course of patients who finally did not undergo EVT after transfer to a CSC has rarely been addressed in the large stroke trials. In our study, the most important reasons for futile transfer were progression of infarct, spontaneous recanalization of the culprit artery, and/or clinical improvement in the stroke deficits.

In comparison to our data, the Madrid stroke network study in Spain had 41% futile transfer [7], while Morey et al. reported 36% futile transfers in their study at Mount Sinai Health System situated in New York [5]. In both these studies, recanalization of the target vessel was the most common reason for not performing EVT after the transfer to an EVT-capable center. The rate of futile transfer was >50% in the study by Yi et al., where patients were transferred based on clinical assessment without the vascular imaging [8]. Futile transfer rate was 45% in a paper by Sablot et al. The study population included semi-rural and mountainous regions in France with relatively longer travel times [9]. Rates of futile transfer were relatively lower in our cohort compared to previous studies, likely due to a combination of reasons, including completion of vascular imaging and confirmation of LVO prior to transfer, urban settings with relatively shorter travel time, improvements in the system of care, improving expertise and patient selection criteria, etc.

Out of the 85 patients who did not have EVT, we found that 44.7% did not have LVO upon transfer. Almost 90% of patients in this subgroup received IVT. This group includes patients who underwent recanalization, either due to IVT or spontaneously. This observation is in line with the studies showing that patients who undergo IVT have a better outcome compared to those who directly undergo EVT [10,11]. In the cohort reported by Morey et al. [5], 31% of patients had no LVO at CSC. However, they included some patients who did not undergo CTA at PSC, and a lower number (64%) of patients in this subgroup received IVT. In a study by Yi et al., where patients were transferred without vascular imaging, 71% patients had no LVO on arrival [8]. Rates of arterial recanalization were >50% in the French study by Sablot et al. However, the study included patients presenting only within 4.5 h of stroke onset and transferred only 52.6% of LVO patients for EVT due to various reasons, including clinical improvement, weather conditions, and unavailability of transport facilities [9].

Progression of infarct led to futility of transfer in 29.4% of the study population, which was similar to Morey et al. (34%) and the Madrid Study (32%) [5,7]. Infarct progression was seen in 21% patients in the French study, where patients were selected only within the early EVT window (<6 h) based on MRI imaging criteria [12]. The rate of futile transfer due to infarct progression was 10% in the study by Yi et al., likely due to a larger denominator, as patients were selected for transfer without the vascular imaging. All patients in our study were transferred based on CT angiography and confirmation of LVO. Patients in this subgroup had higher NIHS scores at the time of presentation to PSC, and deficits persisted upon transfer. The clinical severity of stroke is a marker for tissue injury and correlates with the volume of infarct at that stage. This explains the futility of transfer in this subgroup. ASPECTS decay is a measure of stroke progression. The presence of good collateral circulation may slow down the ASPECTS decay, and help more brain tissue to survive until recanalization with EVT can be achieved [13]. A study by D’Anna et al. found that collateral status is an independent predictor of futile recanalization [14]. The CT angiograms were performed in all cases and were assessed by treating stroke neurologists and neurointerventionists for LVO. However, the study team could not access the scans at all the referring hospitals for detailed analysis of collateral grading. Hence, we could not compare the status of collaterals between the two groups. This is an important limitation of our study.

Delays in the inter-hospital transfer process, including time spent at PSC, remain one of the predominant causes for the infarct progression. Clinical readiness to evaluate patients for potential EVT at PSC also plays an important role. The time it takes for the PSC team to perform necessary imaging and activate the endovascular team can introduce additional delays [15]. Measures such as bypassing the PSC and transferring the patient directly to CSC for IVT and EVT can increase the number of patients who can undergo EVT by reducing the time interval from LKW to treatment. Factors such as the geographical location, round-the-clock availability of the neurointerventional team, and the technical logistics of patient transfer are intertwined with these delays. Innovative models like Mobile Interventional Stroke Teams (MIST) have shown promise in reducing time to EVT [16]. Availability of advanced imaging, including CT perfusion and appropriate collateral scoring, would help in detecting fast progressors who would benefit the most from interfacility transfer. A study by Seners et al. found that high hypoperfusion intensity ratio (HIR) on perfusion imaging, intracranial carotid artery occlusion, and exclusively deep infarct location at the primary center were associated with rapid infarct growth [17]. Furthermore, with the recent data indicating benefits of EVT even in patients with large core infarct or low ASPECT, this subgroup will be considered for EVT more often, and that will further reduce the number of futile transfers [18,19]. There is uncertainty regarding EVT in patients with LVO with low NIHSS (4 or less). These patients may be considered for EVT based on the clinician’s judgment if there is a disabling deficit such as aphasia. At the time of sample collection, results from large core EVT trials were not yet available; therefore, these patients did not undergo EVT.

Our study has several limitations, primarily due to its retrospective design. Additionally, the generalizability of the findings may be restricted to EVT centers in urban areas with relatively short transfer times. It is likely that some patients may have had more than one reason for not undergoing EVT, resulting in some overlap in subgroups. Furthermore, this data is from the time before large core EVT trials. These studies have resulted in the inclusion of more patients for EVT than before, thus reducing the number of futile transfers.

## 5. Conclusions

Our study concluded that 26% of inter-hospital transfers for EVT were futile, primarily due to infarct growth, recanalization of the target vessel, and low NIHSS. Better patient selection, faster transfer to CSC, and developing better systems of stroke triage may help in the reduction of futile inter-hospital stroke transfers.

## Figures and Tables

**Table 1:** Study population characteristics.

Study Population Characteristics	No EVT (*n* = 85)	EVT (*n* = 241)	*p* Value
Age, mean (SD)	71 (12.7)	69.6 (14.3)	0.423
Female, *n* (%)	39 (45.9)	105 (43.6%)	0.712
PSC NIHSS, median (Range)	16 (2–33)	17 (4–36)	0.146
CSC NIHSS, median (Range)	9 (0–33)	17 (4–31)	0.000
IVT at PSC, *n* (%)	64 (75.3)	132 (54.8)	0.001
LKN to PSC in minutes, mean (SD)	231.9 (209.5)	166.2 (221.8)	0.018
DIDO at PSC in minutes, mean (SD)	91.7 (29.2)	105 (43.8)	0.01
Transport time in minutes, mean (SD)	49.8 (18.9)	47.2 (17)	0.246
LKN to CSC in minutes, mean (SD)	373.5 (209.9)	318.4 (230.4)	0.054
ASPECTS at PSC, median (Range)	8 (6–10)	8 (5–10)	0.001
ASPECTS at CSC, median (Range)	6 (5–10)	7 (3–10)	0.004
ASPECTS decay during transport, median (Range)	1 (0–4)	1 (0–5)	0.612

Notes: SD—Standard Deviation, PSC—Primary Stroke Center, CSC—Comprehensive Stroke Center, NIHSS—National Institute of Health Stroke Scale, IVT—Intravenous Thrombolysis, LKN—Last Known Normal, DIDO—Door-In-Door-Out Time, ASPECTS—Alberta Stroke Program Early CT Score.

**Table 2:** Multivariate analysis of variables between the two groups.

Study Population Characteristics	OR	95% Confidence Interval	*p* Value
Age, mean (SD)	1.01	(0.96–1.03)	0.96
Female, *n* (%)	1.14	(0.43–3.01)	0.78
PSC NIHSS, median (Range)	1.01	(0.90–1.13)	0.77
CSC NIHSS, median (Range)	1.04	(0.92–1.17)	0.48
IVT at PSC, *n* (%)	0.69	(0.23–2.0)	0.49
LKN to PSC in minutes, mean (SD)	1.0	(0.99–1.01)	0.34
DIDO at PSC in minutes, mean (SD)	1.0	(0.99–1.02)	0.36
Transport time in minutes, mean (SD)	1.01	(0.97–1.03)	0.77
LKN to CSC in minutes, mean (SD)	0.99	(0.98–1.007)	0.362
ASPECTS at CSC, median (Range)	1.22	(0.86–1.71)	0.25

Notes: OR—Odds Ratio, SD—Standard Deviation, PSC—Primary Stroke Center, CSC—Comprehensive Stroke Center, NIHSS—National Institute of Health Stroke Scale, IVT—Intravenous Thrombolysis, LKN—Last Known Normal, DIDO—Door-In-Door-Out Time, ASPECTS—Alberta Stroke Program Early CT Score.

**Table 3:** Characteristics of futile transfer cohort.

Futile Transfer	Recanalization (*n* = 38)	Established Infarct (*n* = 25)	Low NIHSS (*n* = 15)
PSC NIHSS, median (Range)	13 (2–30)	19 (12–33)	10 (2–20)
CSC NIHSS, median (Range)	5 (0–30)	21 (10–33)	3 (0–9)
IVT at PSC, *n* (%)	34 (89.4)	14 (56)	10 (66.7)
Arrival at CSC within 6 h of LKN, *n* (%)	25 (65.7)	14 (56)	7 (46.7)
LKN to PSC in minutes, mean (SD)	228.8 (204.7)	217.6 (181.8)	279.2 (281.5)
DIDO at PSC in minutes, mean (SD)	89.9 (31.5)	93.5 (22.5)	87.8 (24.3)
Transport time in minutes, mean (SD)	50.7 (19)	48.3 (17.2)	46.8 (12.2)
LKN to CSC in minutes, mean (SD)	369.5 (206.9)	359.5 (178.5)	413.8 (276.7)

Notes: PSC—Primary Stroke Center, NIHSS—National Institute of Health Stroke Scale, CSC—Comprehensive Stroke Center, IVT—Intravenous Thrombolysis, LKN—Last Known Normal, DIDO—Door-In-Door-Out Time.

## Data Availability

The original contributions presented in this study are included in the article. Further inquiries can be directed to the corresponding author.

## Abbreviations

PSC—Primary Stroke Center, CSC—Comprehensive Stroke Center, NIHSS—National Institute of Health Stroke Scale, IVT—Intravenous Thrombolysis, EVT—Endovascular Thrombectomy, LKN—Last Known Normal, DIDO—Door-In-Door-Out Time, ASPECTS—Alberta Stroke Program Early CT Score.
